# Correlation analysis of cardiopulmonary exercise test indices and conditions of overweight patients with obstructive sleep apnea: a retrospective study

**DOI:** 10.1590/1516-3180.2022.0264.R2.010623

**Published:** 2023-10-13

**Authors:** Ying Huang, Chunyan Ruan, Peng Wu, Qian Cai, Yu Chen, Changcai Xie, Jianying Lang, Jiqiang Li, Hai Chen

**Affiliations:** I MSc. Physician, Department 3 of Geriatrics, Guangdong Provincial Hospital of Chinese Medicine, Guangdong Province, China.; II BA. Physician, Department 3 of Geriatrics, Guangdong Provincial Hospital of Chinese Medicine, Guangdong Province, China.; III MSc. Physician, The First Clinical Medical College, Guangzhou University of Chinese Medicine, Guangdong Province, China.; IV MSc. Physician, Department 3 of Geriatrics, Guangdong Provincial Hospital of Chinese Medicine, Guangdong Province, China.; V MSc. Physician, Department 3 of Geriatrics, Guangdong Provincial Hospital of Chinese Medicine, Guangdong Province, China.; VI MD. Physician, Department 3 of Geriatrics, Guangdong Provincial Hospital of Chinese Medicine, Guangdong Province, China.; VII MD. Physician, Guangzhou University of Chinese Medicine, Guangdong Province, China.; VIII MD. Physician, Department 3 of Geriatrics, Guangdong Provincial Hospital of Chinese Medicine, Guangdong Province, China.; IX MSc. Physician, Department 3 of Geriatrics, Guangdong Provincial Hospital of Chinese Medicine, Guangdong Province, China.

**Keywords:** Sleep apnea, obstructive, Overweight, Exercise test, Cardiopulmonary exercise test, Correlation analysis, Condition

## Abstract

**BACKGROUND:**

The cardiopulmonary function of patients with obstructive sleep apnea (OSA) is significantly lower than that of patients with simple snoring and is significantly related to the severity of OSA. Currently, only a few studies have been conducted on cardiopulmonary exercise testing in overweight patients with OSA.

**OBJECTIVE:**

To analyze the correlation between cardiopulmonary exercise test (CPET) indices and the condition of overweight patients with OSA.

**DESIGN AND SETTING:**

Retrospective study in Guangdong Provincial Hospital of Chinese Medicine.

**METHODS:**

This study included 73 hospitalized overweight patients. The patients were divided into no, mild, moderate, and severe OSA groups. Differences in the CPET indices among the four groups were compared. The correlation between the CPET indices and conditions was analyzed.

**RESULTS:**

No, mild, moderate, and severe OSA groups had 18 men and 5 women, 11 men and 3 women, 12 men and 2 women, and 21 men and 1 woman, respectively (P > 0.05). No significant difference was observed in resting pulmonary function among the four groups (P > 0.05). In the CPET, the anaerobic threshold, maximum oxygen uptake, and oxygen pulse were significantly lower in the severe OSA group than those in the normal OSA group (P < 0.05). Moreover, CPET indices negatively correlated with the apnea-hypopnea index.

**CONCLUSION:**

Changes in CPET indices occurred earlier than changes in resting pulmonary function in patients with OSA. CPET might be a potential method for evaluating the severity of OSA combined with overweight status.

## INTRODUCTION

Obstructive sleep apnea (OSA) is sleep-disordered breathing involving respiratory, cardiovascular, neurological, digestive, endocrine, and other systemic systems.^1^ It refers to the repeated complete or partial obstruction of the upper airway during sleep, which causes frequent apnea or reduced ventilation, leading to intermittent hypoxemia, hypercapnia, and sleep structure disorders.^2^ The main clinical manifestations of OSA are snoring with apnea and daytime sleepiness, which can cause damage to multiple organ functions.^3^ Epidemiological surveys show that the prevalence in the middle-aged population and men is 2% and 4%, respectively.^4^ The prevalence of OSA increases with age.^5^

It is reported that the prevalence of OSA in patients with body mass index (BMI) exceeding 30 kg/m^2^ and metabolic syndrome is 40% and 60%, respectively.^6,7^ All of the basic research, epidemiological, and clinical data show that obesity is one of the most important risk factors for OSA, and the incidence rate of OSA is strongly correlated with overweight.^8^ OSA is an independent risk factor for metabolic syndrome, which can be complicated by diabetes, obesity, hyperlipidemia, and other diseases.^9^ Previous studies have confirmed that the cardiopulmonary function of patients with OSA is significantly lower than that of patients with simple snoring and is significantly related to the severity of OSA.^10^ However, there are few studies on cardiopulmonary exercise tests (CPETs) of patients with overweight or obesity combined with OSA.

## OBJECTIVE

This study aimed to explore the correlation between CPET indices and the condition of overweight patients with OSA.

## METHODS

### Subjects

This retrospective study was approved by the Ethics Committee of Guangdong Provincial Hospital of Chinese Medicine (Data: December 20, 2019; Approval number: BF2019-216-01) All the participants provided written informed consent. From January 1, 2018, to December 31, 2019, 73 overweight patients hospitalized at the Guangdong Provincial Hospital of Chinese Medicine were included. The inclusion criteria were as follows: (1) patients met the diagnostic criteria of the guidelines for the diagnosis and treatment of OSA hypopnea syndrome (Basic Edition);^11^ (2) patients who were older than 18 years; (3) patients with BMI ≥ 24 kg/m^2^; (4) patients who underwent CPET to establish exercise-training protocols. The exclusion criteria were as follows: (1) patients who had chronic obstructive pulmonary disease or bronchial asthma; (2) patients who had malignant tumors; (3) patients who had immune system and acute and chronic infectious diseases; (4) patients with hypertension, diabetes, hyperlipidemia, and other basic diseases; (5) patients with severe upper respiratory tract obstruction; (6) patients accompanied by other diseases affecting cardiopulmonary function; and (7) patients who received regular treatment for OSA (such as continuous positive airway pressure [CPAP]).

### Diagnostic criteria

The diagnostic criteria for overweight were as per the health industry standard of the People’s Republic of China – Determination of adult weight formulated in 2013, 18.5 ≤ BMI < 24 kg/m^2^, normal; 24 ≤ BMI < 28 kg/m^2^, overweight; and BMI ≥ 28 kg/m^2^, obese.^12^ The diagnostic criteria of OSA were in accordance with the guidelines for the diagnosis and treatment of OSA hypopnea syndrome (basic level version),^11^ mainly based on the medical history, signs, and polysomnography (PSG) results. OSA can be diagnosed if there are typical symptoms, such as night sleep snoring with apnea, Epworth Sleepiness Scale (ESS) score ≥ 9, stenosis and obstruction of any part of the upper airway, and apnea-hypopnea index (AHI) ≥ 5 times/h. For those whose daytime sleepiness is not obvious (ESS score < 9), OSA can be diagnosed if there is an AHI of > 5 times/h, cognitive impairment, hypertension, coronary heart disease, cerebrovascular disease, diabetes, or insomnia. According to the AHI, the severity classification is divided into mild (5 ≤ AHI < 15 times/h), moderate (15 ≤ AHI < 30 times/h), and severe (AHI ≥ 30 times/h).

### Grouping and general data collection

Based on the AHI, patients were divided into no (n = 23), mild (n = 14), moderate (n = 14), and severe (n = 22) OSA groups. The sex, age, height, and weight of patients were also recorded. The ESS was used to assess excessive daytime sleepiness.

### Sleep breathing monitoring

On the day of the examination, patients were forbidden from drinking coffee or strong tea. On the night of the examination, patients were forbidden from consuming sedatives and sleeping AIDS. An Anbolan M2 sleep-breathing monitor (Anbolan (Beijing) Medical Equipment Co. Ltd., Beijing, China) was used to detect breathing. Oronasal airflow, chest and abdominal movements, finger oxygen saturation, snoring, and pulse rate were recorded. During the monitoring period, the signal was kept in good condition, and the monitoring time throughout the night shall be ≥ 7 h. A report was generated after a review by a sleep-monitoring technician and a sleep professional physician. Detection indicators included the apnea-hypopnea index (AHI, times/h), minimum blood oxygen saturation (%), average blood oxygen saturation (%), percentage of sleep time with blood oxygen saturation < 90% of the total sleep time (TS90%), and oxygen reduction index (times/h).

### Cardiopulmonary exercise test

First, resting pulmonary function was tested. The percentage of forced expiratory volume in the predicted value in the first second (FEV1%), percentage of forced expiratory volume in the predicted value (FVC%), one-second rate ((FEV1/FVC) %), percentage of maximum mid-expiratory flow in the predicted value, and percentage of maximum ventilation in the predicted value in a minute (MVV%) were recorded. The determination was repeated three times, the error value between the two replicates was less than 5%, and the highest value was used for analysis.

After resting for 10 min, a CPET was performed with an increasing exercise load plan. The exercise started with zero load, and the load was gradually increased after 3 min. The power load plan increased by 10-25 W/min. The speed of the bicycle was 60 rpm, and the pedaling time was controlled within 8-12 min. The power load was stopped when there was significant fatigue, shortness of breath, leg fatigue or discomfort, inability to maintain a stable speed, or significant changes in the electrocardiogram. In addition, 0 W power was used to relax for 5 min (i.e., the recovery period), and the exercise test was ended.

### Endpoint

Various parameters were recorded, including the anaerobic threshold (AT, L/min), the percentage of anaerobic threshold in the predicted value (AT/Ref, %), the percentage of maximum oxygen uptake in the predicted value (O_2_ max/PRED, %), respiratory exchange rate (RER), oxygen pulse (O_2_ pulse, ml/beat), the percentage of oxygen pulse in the predicted value (O_2_ pulse), maximum respiratory times (f-ergo max, times/min), respiratory reserve (BR, %), and carbon dioxide ventilation equivalent (EQCO_2_).

### Statistical analysis

SPSS software (version 25.0; International Business Machines Corp., Armonk, New York, United States) was used for the statistical analysis. If the measurement data met the normal distribution, the means ± standard deviation was used for the description; if the measurement data did not meet the normal distribution, the median (interquartile range) was used for the description. Count data are presented as percentages (%). When the quantitative data met the normal distribution and homogeneity of variance criteria, a one-way analysis of variance was used for multigroup comparisons, and the Student-Newman-Keuls (SNK) test was used for pairwise comparisons. When the quantitative data did not meet the normal distribution or homogeneity of variance criteria, the rank sum test was used for multigroup comparisons and the SNK test was used for pairwise comparisons. The chi-square test was used to compare multiple groups of count data. The Pearson product-moment correlation was used to analyze the correlation between exercise cardiopulmonary function and AHI in patients with OSA. P*<*0.05 indicated that the difference was statistically significant.

## RESULTS

### Comparison of general information

The no, mild, moderate, and severe OSA groups comprised 18 men and 5 women, 11 men and 3 women, 12 men and 2 women, and 21 men and 1 woman, respectively (P > 0.05). The age in the no, mild, moderate, and severe OSA groups were 50.00 ± 11.236, 51.00 ± 8.218, 54.00 ± 12.134, and 47.64 ± 6.268, respectively (P > 0.05). No significant differences were observed in the proportion of men and women, age, BMI, or other general characteristics among the four groups (P > 0.05, **Table 1**).

**Table 1.** Comparison of general information for groups

Groups	Cases	Gender		Age (years old)	BMI
		Male	Female		
no OSA group	23	18	5	50.00 ± 11.24	27.70 ± 2.43
Mild OSA group	14	11	3	51.00 ± 8.22	26.85 ± 2.88
Moderate OSA group	14	12	2	54.00 ± 12.13	27.30 ± 4.51
Severe OSA group	22	21	1	47.64 ± 6.27	28.55 ± 4.43
χ^2^ or F			χ^2^ = 3.152	*F* = 1.278	χ^2^ = 4.122
P		0.369		0.289	0.249

OSA = obstructive sleep apnea; BMI = body mass index.

### Comparison of sleep monitoring and pulmonary functions

Sleep monitoring showed that the ESS, oxygen reduction index, minimum oxygen saturation, average oxygen saturation, TS90%, and MVV were significantly different among the four groups (P < 0.001 or P < 0.05). Moreover, significant differences were observed in the minimum oxygen saturation, mean oxygen saturation, and TS90% between the severe OSA group and the other three groups (P < 0.05). Similarly, significant differences were noticed in the minimum and average oxygen saturations between the moderate and no OSA groups (P < 0.05, **Table 2**). 

**Table 2.** Comparison of polysomnography and pulmonary functions

Items	no OSA group	Mild OSA group	Moderate OSA group	Severe OSA group	χ^2^ or F	P values
ESS	1.0 (0.00, 2.00)	5.5 (3.00, 7.25)	9.0 (6.75, 11.25)	11.0 (9.00, 16.00)	53.642	< 0.001^*^
ORI (times/h)	2.90 (1.10, 4.10)	10.60 (6.65, 15.45)	25.35 (20.85, 28)	44.15 (35.33, 68.3)	63.721	< 0.001^*^
MOS (%)	89.0 (85.00, 90.00)	86.0 (79.00, 87.25)	80.0 (78.50, 84.00)^d^	69.5 (61.25, 76.00)^a b c^	46.577	< 0.001^*^
AOS (%)	95.40 (94.20, 96.40)	94.70 (93.78, 95.80)	93.50 (92.98, 95.03)^d^	91.25 (88.78, 93.43)^a b c^	34.949	< 0.001^*^
TS90 (%)	0.05 (0.00, 0.20)	0.75 (0.18, 2.15)	4.10 (2.25, 8.45)	23.75 (11.13, 47.40)^a b c^	54.958	< 0.001^*^
FEV1 (%)	84.70 ± 16.85	80.43 ± 18.79	91.14 ± 14.49	81.27 ± 12.72	1.446	0.237
FEV1/FVC (%)	83.87 ± 9.27	85.07 ± 9.45	85.21 ± 7.43	84.77 ± 8.22	0.094	0.963
FVC (%)	89.0 (70.00, 94.00)	82.5 (62.50, 92.25)	85.5 (78.25, 94.00)	81.0 (71.00, 91.00)	1.965	0.580
MMEF (%)	84.39 ± 26.03	76.29 ± 24.34	85.93 ± 23.71	79.14 ± 22.90	0.545	0.653
MVV (%)	106.57 ± 18.61	94.86 ± 26.90	109.21 ± 22.53	90.41 ± 20.50	3.242	0.027^**^

^*^ P < 0.05

^a^ P < 0.05, compared with no OSA group

^b^ P < 0.05, compared with mild OSA group

^c^ P < 0.05, compared with moderate OSA group

^d^ P < 0.05, compared with no OSA group

^**^ P < 0.05, there was no significant difference between the four groups by Student–Newman–Keuls method.

OSA = obstructive sleep apnea; ESS = Epworth Sleepiness Scale; ORI = oxygen reduction index; MOS = minimum oxygen saturation; AOS = average oxygen saturation (%); TS90 = < 90% in total sleep time; FEV1 = forced expiratory volume in the predicted value in the first second; FVC = forced expiratory volume in the predicted value; MMEF = maximum mid expiratory flow; MVV = maximum ventilation in the predicted value in a minute.

### Comparison of exercise cardiopulmonary test indexes

Differences were observed in O_2_max/PRED%, AT/Ref%, and O_2_pulse% among the four groups (P < 0.05). Specifically, O_2_max/PRED%, AT/Ref%, and O_2_pulse% were significantly different between the overweight and severe OSA groups (P < 0.05). However, no marked differences were noticed in O_2_max/PRED%, AT/Ref%, or O_2_pulse% between the other two groups (P > 0.05; **Table 3**).

**Table 3.** Comparison of cardiopulmonary exercise test indexes

Items	no OSA group	Mild OSA group	Moderate OSA group	Severe OSA group	χ^2^ or F	P values
AT, l/min	16.10 (11.60, 20.70)	12.10 (9.88, 16.83)	13.15 (10.90, 19.30)	12.25 (10.15, 14.15)	7.655	0.054
O_2_max/pred%	88.65 ± 13.52	75.57 ± 17.32	78.14 ± 15.63	72.41 ± 21.06^a^	*3*.685	0.016^*^
AT/Ref%	60.70 ± 316.43	47.21 ± 16.58	51.71 ± 18.41	44.13 ± 10.38^a^	4.823	0.004^*^
RER	1.24 ± 0.19	1.18 ± 0.15	1.17 ± 0.16	1.20 ± 0.11	0.848	0.472
O_2_ pulse ml/beat	10.80 (8.40, 13.70)	9.05 (8.08, 9.95)	9.95 (7.40, 12.70)	9.10 (7.75, 10.83)	3.754	0.289
O_2_ pulse%	80.95 ± 12.69	75.14 ± 17.25	74.07 ± 15.81	66.77 ± 18.92^a^	2.879	0.042^*^
F-ergo (max time/min)	34.95 ± 6.98	32.14 ± 4.85	31.35 ± 5.72	31.81 ± 5.49	1.541	0.211
BR%	47.56 ± 12.68	55.00 ± 13.92	58.93 ± 11.85	49.54 ± 14.41	2.589	0.060
EQCO_2_	26 (23.00, 27.00)	25 (23.00, 27.25)	25 (23.50, 26.00)	25 (22.00, 26.25)	1.240	0.743

^*^ P < 0.05

^a^ P < 0.05, compared with no OSA group.

OSA = obstructive sleep apnea; AT = anaerobic threshold; RER = respiratory exchange rate; 0_2_= oxygen; BR% = respiratory reserve; EQCO_2_= carbon dioxide ventilation equivalent.

### Correlation analysis between AHI and exercise cardiopulmonary test indexes

The severity of OSA is generally expressed by the AHI. Pearson correlation analysis showed that OSA severity was negatively correlated with AT, AT/Ref%, O_2_max/PRED, and O_2_ pulse (P < 0.05). However, OSA severity of OSA was not correlated with RER or EQCO_2_ (P > 0.05, **Table 4**).

**Table 4.** Correlation analysis between AHI and exercise cardiopulmonary test indexes.

Items	AHI	
	Correlation coefficient (r)	P values
AT, l/min	-0.273	0.019^*^
O_2_max/PRED%	-0.251	0.032^*^
AT/Ref%	-0.295	0.011^*^
RER	-0.015	0.899
O_2_ pulse ml/beat	-0.119	0.318
O_2_ pulse%	-0.301	0.01^*^
EQCO_2_	-0.171	0.148

^*^ P* *< 0.05.

AHI = apnea-hypopnea index; AT = anaerobic threshold

RER = respiratory exchange rate; 0_2_= oxygen; BR% = respiratory reserve

EQCO_2_= carbon dioxide ventilation equivalent.

## DISCUSSION

In the present study, we revealed that changes in CPET indices occurred earlier than changes in resting pulmonary function in patients with OSA. CPET might be a potential method for evaluating the severity of OSA combined with overweight status.

OSA is a chronic disease with multiple system damage.^13^ The clinical manifestations of mild or early OSA are often hidden.^14^ When it develops from moderate to severe, it causes irreversible damage to the body, thus losing the best opportunity for treatment.^15,16^ Therefore, it is of positive clinical significance to accurately assess the severity of patients with OSA. PSG or sleep outside center monitoring (OCST) is the gold standard for the diagnosis of OSA.^17,18^ The AHI measured using PSG or OCST is the most important indicator for evaluating the degree of obstruction. However, studies have found that AHI does not truly reflect the severity of the condition.^19^ For example, for patients with mild to moderate OSA, even if the AHI level is the same, the severity of hypoxemia and arousal can be quite different.^20^ Moreover, the results of a 2008 study on the cardiovascular endpoint events of sleep apnea (Sleep Apnea Cardiovascular Endpoints study) showed that for patients with OSA and cardiovascular disease, after CPAP treatment, although the AHI index of the patients can be reduced and hypoxia can be improved, it does not affect the cardiovascular risk.^21^ It is suggested that a single AHI cannot be used as a predictor of cardiovascular events in patients with OSA.

The CPET mainly relies on exercise stress and comprehensively detects changes in oxygen uptake and carbon dioxide emissions in the heart and lungs under different loads and electrocardiograms. CPET helps to reflect the degree of exercise restriction.^22^ The potential of cardiopulmonary function can be evaluated by CPET.^23^ Moreover, it can formulate individualized intensity exercise programs to meet the needs of patients with different needs for disease rehabilitation.^24^ As a noninvasive, safe, and simple detection method,^25^ CPET has not been popularized in China, and the evaluation value of various indicators for OSA has not been fully agreed upon.

Although some studies have shown that AHI, the most important index reflecting disease severity, may not necessarily correlate with the degree of nocturnal hypoxia and the lethargy scale score.^26^ In this study, according to the comparison of symptoms, hypoxia, and other indicators among the four groups, symptoms and hypoxia were more serious with an increase in AHI. For example, there were significant differences in the oxygen reduction index and ESS scores between the groups. There were marked differences in the minimum oxygen saturation, average oxygen saturation, and TS90% between the severe OSA group and the other three groups. These results showed that AHI had a good correlation with the sleepiness scale score and hypoxemia, which may be related to the fact that the patients were overweight. This suggests that in overweight and OSA patients, the symptoms and degree of hypoxia become increasingly serious with the progression of the disease.^27^ There was no significant difference in resting static pulmonary function among the four groups, indicating that resting static pulmonary function has limitations in evaluating OSA severity. There were significant differences in MVV among the four groups, but there was no significant difference among the four groups using the SNK method, which is consistent with previous literature reports.^28^ The contradiction may be related to the small sample size.

In the CPET, the anaerobic threshold, maximum oxygen uptake, and oxygen pulse in the severe OSA group were significantly lower than those in the no OSA group and negatively correlated with AHI. This suggests that the anaerobic threshold, maximum oxygen uptake, and oxygen pulse decreased with disease aggravation, especially in the severe OSA group. The anaerobic threshold refers to the maximum oxygen uptake value when a patient’s aerobic function does not require the supplementary function of anaerobic metabolism during exercise. This was the highest oxygen uptake observed in the absence of lactic acidosis. It represents the ability of the circulatory system to transport oxygen and reflects a patient’s cardiac function. The maximum oxygen-carrying capacity reflects the blood pumping limit of the heart and the oxygen uptake capacity of sports tissues.^29^ Oxygen pulse reflects the level of cardiac output and cardiac reserve capacity and is a main index of cardiopulmonary function under maximum load.^30^ The above results suggest that the changes in cardiopulmonary exercise test indexes in overweight OSA patients occur earlier than resting static pulmonary functions. CPET can be used as an auxiliary method to evaluate the severity of OSA in overweight patients.

Furthermore, there is poor compliance with the traditional treatment of OSA, such as noninvasive positive pressure ventilation.^31^ The CPET is used to understand the cardiopulmonary function of overweight patients with OSA. Early intervention for patients with a downward trend in cardiopulmonary exercise indicators can prevent disease progression. In this study, there were no significant differences in CPET indices between the mild OSA, moderate OSA, and no OSA groups. Moreover, there were no significant differences in respiratory reserve and carbon dioxide ventilation equivalents among the four groups. This may be due to small sample sizes.

## CONCLUSION

In conclusion, the CPET may be a potential method for assessing the severity of OSA and overweight status. It provides clinical evidence for formulating exercise prescriptions and early weight loss interventions, which is of great significance in preventing disease exacerbation and improving prognosis.
